# RNU6B, a frequent reference in miRNA expression studies, differentiates between deaths caused by hypothermia and chronic cardiac ischemia

**DOI:** 10.1007/s00414-019-02041-0

**Published:** 2019-03-23

**Authors:** Helena Kaija, Lasse Pakanen, Katja Porvari

**Affiliations:** 1 Faculty of Medicine, Research Unit of Internal Medicine, Department of Forensic Medicine, Medical Research Center Oulu, Oulu University Hospital and University of Oulu, P.O. Box 5000, FI-90014 Oulu, Finland; 2 Forensic Medicine Unit, National Institute for Health and Welfare, P.O. Box 310, FI-90101 Oulu, Finland; 3 Faculty of Medicine, Research Unit of Cancer Research and Translational Medicine, Department of Pathology, Medical Research Center Oulu, Oulu University Hospital and University of Oulu, P.O. Box 5000, FI-90014 Oulu, Finland

**Keywords:** Reference miRNAs, RNU6B expression, Human post mortem heart samples, Hypothermia deaths, Chronic cardiac ischemia deaths

## Abstract

Here, we tested the usefulness of small non-coding RNAs as references in quantitative RT-PCR expression analyses in hypothermia and chronic cardiac ischemia as the primary causes of death. Cq values of RNU6B, SCARNA17, SNORD25, and SNORA73A were determined from human cadaver samples of hypothermia and cardiac deaths. Average Cq values of RNU6B were higher in hypothermic and average SCARNA17 Cq values in chronic ischemic samples, but no difference in SNORD25 and SNORA73A Cq values could be seen between the groups. RNU6B expression levels were calculated using SNORD25, SNORA73A, and their combination as the reference in normalization. Expression of RNU6B, a widely used reference, was found to be significantly lower in hypothermia than in chronic cardiac ischemia. In these conditions, RNU6B is a useful marker differentiating hypothermia deaths from chronic ischemic heart disease deaths, but not a valid reference for normalization in expression studies.

## Introduction

The expression of genes is influenced by various physiological and pathological conditions such as hypothermia and myocardial ischemia. Small non-coding RNAs (small ncRNAs) participate in the regulation of gene expression at post-transcriptional level. Small ncRNAs (length less than 400 nucleotides) are a versatile class of non-coding RNAs including, among others, microRNAs (miRNA), small nucleolar RNAs (snoRNA and scaRNA), and spliceosomal RNAs (RNU) [1].

Molecular level changes elucidate different death processes in general and have potential impact on death investigations in the future [2]. In routine forensic pathology, mRNA analyses could be used in wound and injury age determination [3]. Like gene expression, the expression of small ncRNAs is variable as well. These changes, especially those of miRNA levels, are typically utilized in forensic body fluid and tissue identification [4, 5] and in cancer diagnostics and prognostic evaluation [6].

The quality of RNA is variable in post mortem samples. RNA integrity is considered more critical than post mortem interval (PMI) for defining suitability of tissue for molecular biology applications [7, 8]. The ribosomal-based methods of RNA evaluation scale the sample quality from 1 (degraded) to 10 (intact) [9]. The RNA integrity values RIN (RNA integrity number, Agilent Technologies, Santa Clara, USA) and RIS (RNA integrity score, Qiagen, Hilden, Germany) have been shown to be comparable in heat-degraded and RNAase-degraded RNA [10]. However, the relative expression values determined using short amplicons with typical length of 70–250 bp are practically independent of the RNA quality in qPCR method [11].

Coronary artery disease and signs of chronic myocardial ischemia are frequent findings in medico-legal autopsies in the western population [12]. Our aim was to test the suitability of cadaver myocardial expression levels of small ncRNAs RNU6B, SCARNA17, SNORD25, and SNORA73A as references when hypothermia deaths and cardiac deaths are investigated.

## Materials and methods

### Study material

The study cases were chosen on the bases of autopsy reports and the causes of death. The total number of hypothermia cases in this study was 25: 20 of them having hypothermia as the primary cause of death (group H) and 5 as a contributing cause of death (group cH). Of the 44 ischemic heart disease cases, 17 had atherosclerotic heart disease as the primary cause of death by itself (group AHD) and 27 with acute myocardial infarction as direct cause of death (group AHD + AMI).

The permissions to use cadaver tissue samples and data from medico-legal death investigations were granted by the National Supervisory Authority for Welfare and Health (Dnro 2932/06.01.03.01/2013) and the National Institute for Health and Welfare (Dnro THL/479/5.05.01/2013), respectively. The Northern Ostrobothnia Hospital research ethics committee approved the study protocol (31/2007).

Fresh, non-formalin-fixed tissue samples from the anterior wall of the left ventricle were collected for research purposes in medico-legal autopsies in the Department of Forensic Medicine, University of Oulu, Finland, and the National Institute for Health and Welfare, Oulu, Finland. The samples were stored at − 80 °C.

### cDNA synthesis and real-time quantitative PCR

A miScript II Reverse Transcription Kit (Qiagen, Hilden, Germany) was used for the synthesis of cDNAs from previously extracted (miRNeasy kit) and quality checked (RIS) RNA.

Four small ncRNAs were randomly selected from a miScript PCR Control Set kit (Qiagen) recommended to be used in miRNA studies. miScript SYBR Green PCR kit (Qiagen) and miScript Primer Assays (Qiagen) were used for RNU6B, SCARNA17, SNORD25, and SNORA73A amplifications (< 250 bp) with a Rotor Gene Q (Qiagen) instrument. The RNU6B Cq values were normalized to the Cq values of SNORD25, SNORA73A, and their average value. cDNA from commercial Human Heart Total RNA (Clontech/Takara, Mountain View, CA, USA; RNA pooled from 10 male/female Caucasians, age range 25–51, cause of death trauma) was used as reference sample to calculate fold changes of relative expressions for RNU6B using the 2^-ΔΔCq^ method [13]. The expression levels of the trauma death reference were given the value 1.

### Statistical analysis

Statistical analyses were performed with IBM SPSS statistics, version 24 (Armonk, NY, USA). The hypothermia death groups were compared with the ischemic heart disease death groups. The SPSS tests Shapiro Wilk and Kolmogorov-Smirnov were used to test the normality of the data. Only part of the data followed normal distribution. In addition, the groups were small. For these reasons, the pair-wise comparisons between the groups were made with the non-parametric Mann-Whitney test (statistical significance level set at *p* < 0.05).

## Results

### Amplifications and real-time quantitative PCR

The average Cq values with standard errors of RNU6B, SCARNA17, SNORD25, and SNORA73A as well as the statistical differences between the groups of each variable are presented in Table 1.

**Table 1:** qPCR and RNA integrity data

Group^1^	RNU6B^2^	SCARNA17^2^	SNORD25^2^	SNORA73A^2^	SNORD25 + SNORA73A^2^	RIS^3^
H	18.02 ± 0.41	17.02 ± 0.42	17.87 ± 0.38	17.43 ± 0.57	17.65 ± 0.42	5.20 ± 0.34
cH	16.41 ± 0.61	16.46 ± 0.32	17.01 ± 0.48	16.23 ± 1.00	16.62 ± 0.70	5.96 ± 0.27
AHD	14.13 ± 0.22***, ††	18.68 ± 0.34**, ††	17.72 ± 0.60	18.12 ± 0.66	17.92 ± 0.52	3.96 ± 0.23 **, †††
AHD + AMI	14.30 ± 0.13***, ††	19.30 ± 0.30***, †††	17.80 ± 0.56	16.30 ± 0.58	17.05 ± 0.37	3.56 ± 0.20***, †††

***p < 0.001, **p < 0.01 compared with H; †††*p* < 0.001, ††*p* < 0.01 compared with cH

^1^Hypothermia deaths: H, hypothermia as the main cause of death and cH, hypothermia as a contributing cause of death. Ischemic heart disease deaths: AHD, atherosclerotic heart disease as the primary cause of death by itself and AHD + AMI, atherosclerotic heart disease with acute myocardial infarction

^2^Average Ct values of cardiac small ncRNAs RNU6B, SCARNA17, SNORD25, and SNORA73A with standard errors and statistical differences between the groups

^3^Average RIS values of the samples with standard errors and statistical differences between the groups

The Cq values of RNU6B were higher in both hypothermia groups than those detected in chronic ischemic heart disease. The Cq values of SCARNA were the opposite. No significant differences were detected between the groups in the Cq values of SNORD25 and SNORA73A.

**Fig. 1:**
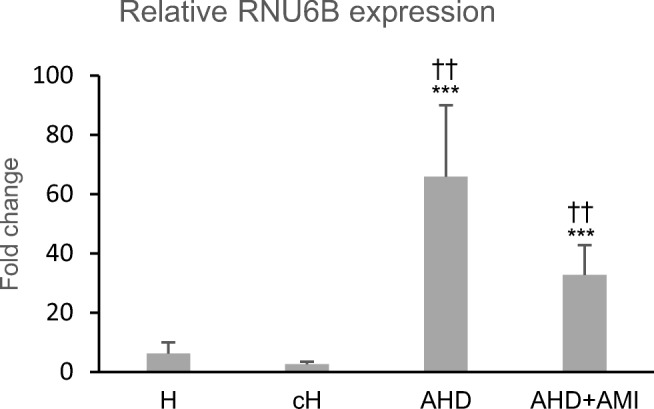
Relative myocardial expression levels of RNU6B in hypothermia and chronic ischemic heart disease. Expression levels were normalized by combination of SNORD25 and SNORA73A. The average fold changes, compared to RNU6B expression in trauma death reference sample, are shown with standard errors. Statistical differences between the groups (H, hypothermia; cH, contributing hypothermia; AHD, atherosclerotic heart disease; AHD + AMI, AHD with acute myocardial infarction): ***p < 0.001 compared with H, †††*p* < 0.001, ††*p* < 0.01 compared with cH

The relative expression levels of RNU6B were analyzed using SNORD25 and SNORA73A either individually (results not shown) or combined (Fig. 1) in normalization. The average fold changes of relative myocardial expression levels of RNU6B were significantly lower in hypothermia than those in chronic ischemic heart disease with or without AMI using all chosen normalizers.

### Correlations between RIS and Ct values

The average RIS values with standard errors and the statistical differences between the groups are presented in Table 1. RIS values correlated with the Cq values of small ncRNAs in the group where hypothermia was a contributing cause of death (RNU6B: *r* = − 0.881, *p* = 0.048; SCARNA17: *r* = − 0.984, *p* = 0.003; SNORA73A: *r* = − 0.969, *p* = 0.006 and the average value of SNORD25 and SNORA73A: *r* = − 0.959, *p* = 0.010).

## Discussion

Small ncRNAs, particularly miRNAs, have lately been frequently evaluated as markers in forensic body fluid identification [4]. Their tissue-specific expression patterns and changeable expression levels in different physiological and pathological conditions make them useful as markers for diagnostic purposes, but also in clarifying mechanisms in response to physiological changes.

RNUs function as guides in the spliceosome formation and intron removal from mRNA [1]. RNUs are widely used for miRNA expression normalization [5, 14]. However, the suitability of the normalizers depends on the conditions and the tissues, and should always be tested [15]. Although the expression of RNUs has been found to be prone to variation in varying conditions, RNU6B has proved to be more stable than many other small ncRNAs in tumor pathology [16]. A reliable reference is needed also in hypothermia research, since cold temperature is known to alter gene expression levels [17–20]. We found Cq values of RNU6B to be significantly higher in hypothermia than in chronic ischemic heart disease in the myocardium. The results were the opposite in SCARNA17 Cq values: this could be because of the better quality of RNA in hypothermia cases, as reflected by their higher RIS values. However, the correlations of RIS with Cq values could only be seen in those hypothermia cases where hypothermia was a contributing cause of death. The stable Cq levels of SNORD25 and SNORA73A suggest their usability for normalization in these conditions.

When the RNU6B expression was determined in the myocardium, normalization with SNORD25, SNORA73A, or their combination gave comparable results. In this study, the myocardial RNU6B expression levels seemed to be higher in hypothermia than the level of the trauma death reference. However, a much striking elevation in the expression levels was seen in chronic cardiac ischemia, possibly due to adaptation mechanisms activating spliceosome formation and intron removal in this chronic condition.

Our results show that small ncRNA RNU6B is not suitable for a reference in cadaver myocardial expression studies of hypothermia and chronic cardiac ischemia. Instead, RNU6B acts as a differentiating marker under these conditions.
